# Household food group expenditure patterns are associated with child anthropometry at ages 5, 8 and 12 years in Ethiopia, India, Peru and Vietnam

**DOI:** 10.1016/j.ehb.2017.02.001

**Published:** 2017-08

**Authors:** Debbie L. Humphries, Kirk A. Dearden, Benjamin T. Crookston, Tassew Woldehanna, Mary E. Penny, Jere R. Behrman, on behalf of The Young Lives Determinants Consequences of Child Growth Project team

**Affiliations:** a Department of Epidemiology of Microbial Disease, Yale School of Public Health, P.O. Box 208034, New Haven, CT 06520-8034, USA; b Department of Global Health, Boston University, Boston, MA, USA; c Department of Health Science, Brigham Young University, Provo, UT, USA; d Department of Economics, Addis Ababa University, Addis Ababa, Ethiopia; e Instituto de Investigación Nutricional, Lima, Peru; f Departments of Economics and Sociology, University of Pennsylvania, Philadelphia, PA, USA

**Keywords:** BMI-Z, body mass index-for-age z score, FAO, Food and Agricultural Organization, HAZ, height-for-age z score, WAZ, weight-for-age z score, WHO, World Health Organization, WHZ, weight-for-height z score, y, year(s), YL, Young Lives study, Household food expenditures, Child growth, Weight gain, Longitudinal cohort study, Household food purchasing patterns

## Abstract

Population-level analysis of dietary influences on nutritional status is challenging in part due to limitations in dietary intake data. Household expenditure surveys, covering recent household expenditures and including key food groups, are routinely conducted in low- and middle-income countries. These data may help identify patterns of food expenditure that relate to child growth.

**Objectives:**

We investigated the relationship between household food expenditures and child growth using factor analysis.

**Methods:**

We used data on 6993 children from Ethiopia, India, Peru and Vietnam at ages 5, 8 and 12y from the Young Lives cohort. We compared associations between household food expenditures and child growth (height-for-age z scores, HAZ; body mass index-for-age z scores, BMI-Z) using total household food expenditures and the “household food group expenditure index” (HFGEI) extracted from household expenditures with factor analysis on the seven food groups in the child dietary diversity scale, controlling for total food expenditures, child dietary diversity, data collection round, rural/urban residence and child sex. We used the HFGEI to capture households’ allocations of their finances across food groups in the context of local food pricing, availability and pReferences

**Results:**

The HFGEI was associated with significant increases in child HAZ in Ethiopia (0.07), India (0.14), and Vietnam (0.07) after adjusting for all control variables. Total food expenditures remained significantly associated with increases in BMI-Z for India (0.15), Peru (0.11) and Vietnam (0.06) after adjusting for study round, HFGEI, dietary diversity, rural residence, and whether the child was female. Dietary diversity was inversely associated with BMI-Z in India and Peru. Mean dietary diversity increased from age 5y to 8y and decreased from age 8y to 12y in all countries.

**Conclusion:**

Household food expenditure data provide insights into household food purchasing patterns that significantly predict HAZ and BMI-Z. Including food expenditure patterns data in analyses may yield important information about child nutritional status and linear growth.

## Highlights

• Exploratory factor analysis identifies a single factor from household food expenditure data.
• We have called this factor the household food group expenditure index.
• Household food group expenditure patterns are associated with significant increases in child HAZ.

## 1 Introduction

Globally, 165 million children are stunted and 50 million children are wasted (Black et al., 2013a). Stunted and wasted children suffer short- and long-term consequences (Walker et al., 2005, Crookston et al., 2011, Victora et al., 2008, Behrman, 2014); therefore, improving children’s nutrition is a global priority (United Nations, 2014, United Nations, 2016). Food intake is one of the causes of undernutrition. It is difficult to identify through population-level analyses what aspects of food intake drive poor nutritional status, in part because information on influences on the food choices that determine consumption is often lacking. Heterogeneities in food consumption may be considerable across households because of variations in preferences, food prices, food availabilities and resource constraints. Investigating patterns in food expenditures at the household level may provide a novel tool for assessing child and household nutritional risk. Furthermore, accurate quantitative measures of dietary intake are time-consuming to obtain, and require extensive food composition databases and nutritional expertise for data collection and analysis (Willett, 1998, Gibson, 2005, Magarey et al., 2011, Fiedler et al., 2013). Additionally, when researchers, program planners and evaluators collect data on foods and liquids consumed in the previous 24 h, this information may not reflect usual intake (Sempos et al., 1985). Because policy makers lack access to information on usual patterns of dietary intake, it is challenging to determine best approaches for improving individuals’ consumption of food.

Data on household food expenditures (defined as market purchases, gifts and foods drawn from own production or stocks consumed by the household) reflect periods longer than 24 h (often 2 weeks) and are consistent, for example, with Living Measurement Surveys conducted by The World Bank and national statistical bureaus (Long et al., 2007). Field workers collecting household expenditure data need to be trained. Similarly, individuals who collect and analyze data using 24-h recalls need specialist training. However, in contrast to use of expenditure data, analysis of dietary intake data requires regular, time-consuming updates of food data bases with information on food preparations, corrections for cooking, waste and portions (Gibson, 2005). Additional advantages and disadvantages of 24-h recall data and expenditure data are outlined in Box 1.

Box 1 **Advantages and disadvantages of dietary intake data relative to household food expenditures data.**

Alt-text: Box 1

Many governmental and non-governmental organizations conduct household consumption and expenditure surveys (HCES) that include information on how much households spend on key food groups. They conduct these surveys every 3–5 years in more than 125 low- and middle-income countries (Fiedler et al., 2012a). Several studies demonstrate significant relationships between nutrient intake based on dietary intake and nutrient intake derived from food expenditures (Naska et al., 2001, Jariseta et al., 2012) after converting food expenditure data to estimates of individual dietary intake (Fiedler et al., 2012a). The HCES surveys are important sources of information on family food choices. Some researchers have converted HCES data to estimates of dietary intake to identify implications for nutritional policy and planning (Jariseta et al., 2012, Fiedler et al., 2012b, Bermudez et al., 2012). One of the important benefits of utilizing HCES data is the lower cost of data collection. According to one estimate, the cost of collecting and analyzing 24-h recall data is estimated to be 75 times that of utilizing HCES data on household food expenditures (Fiedler et al., 2013). We hypothesize that household dietary patterns, as reflected in food expenditure data, are important drivers of family and individual dietary quality which will be manifested in measures of child growth.

Household food expenditure surveys have been used to explore determinants of child nutritional status (Bermudez et al., 2012, Campbell et al., 2010, Sari et al., 2010, Torlesse et al., 2003, Mauludyani et al., 2014). Associations have been observed between specific food expenditure patterns and nutritional status. For example, in Indonesia, Sari and colleagues documented that higher household expenditures on non-grain and animal-source foods reduced the risk of stunting among children ages 0–59 months (Sari et al., 2010). In these studies, researchers used information on expenditures for specific food groups and combinations of food groups, selecting those food groups that have been shown most frequently to be associated with nutritional status.

**Fig. 1:**
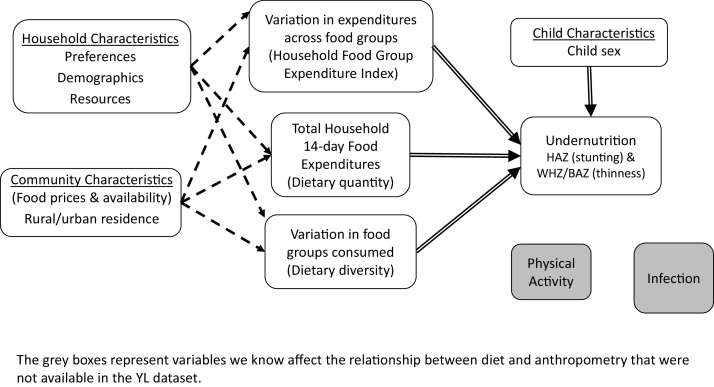
Conceptual Framework.

Data reduction approaches such as factor analysis and principal components analysis (PCA) have been used by some investigators to identify patterns in food consumption based on dietary intake data (Becquey et al., 2010, Bedard et al., 2015, Devlin et al., 2012, Moskal, 2014, Pisa et al., 2015). We extend the use of data reduction approaches to household food expenditures to determine whether there are underlying drivers of food expenditure patterns that are associated with child growth. Fig. 1 represents the conceptual framework guiding this research. We posit that household characteristics (preferences, resources, demographics) and community characteristics (food prices and availability, urbanization) underlie three important indicators of food consumption: 1) allocation patterns of household expenditures across food groups, 2) dietary diversity (an indicator of likelihood of achieving necessary micronutrient intakes), and 3) total household food expenditures (an indicator of dietary quantity). We test the hypotheses that each of these three is associated with children’s nutritional status.

In this study, we examine a cohort of children from four diverse low- and middle-income countries (Ethiopia, India, Peru and Vietnam) using food group expenditure data and a variety of analytic strategies. We characterize household food group expenditure patterns and estimate their associations with children’s nutritional status ((height-for-age (HAZ) and body mass index (BMI-Z)) during early and middle childhood. Our analyses are innovative because 1) our data cover children from ages 5–12 years, 2) we assess the broad applicability of findings across four diverse settings, and 3) we describe associations between food group expenditure patterns and children’s nutritional status. This work contributes to the ongoing conversation about using HCES data for nutrition policy (Fiedler et al., 2012a, Fiedler, 2013), while also exploring a novel approach to identifying patterns in HCES data that is less expensive and less dependent on assumptions than converting HCES expenditure data to estimates of individual level dietary intake.

## 2 Methods

### 2.1 Study design and participants

We used data from the Young Lives (YL) study younger cohort, which is comprised of ∼8000 children in Ethiopia, India, Peru, and Vietnam. The YL study team recruited ∼2000 children from each country in 2002 (round 1) at approximately 1y of age with subsequent data collection at age 5y (round 2: 2006), age 8y (round 3: 2009) and age 12y (round 4: 2013). YL used a multistage sampling design which was pro-poor, with the first stage consisting of selection of 20 sentinel sites. In Ethiopia, the sampling universe included the most food-insecure areas. In Peru, the richest 5% of districts were excluded from the sample. While poor clusters were moderately oversampled, the final samples represented a variety of social, geographic, and demographic groups. The sample in India consisted of households from Andhra Pradesh and Telangana, while the three other countries used nationwide samples (Humphries et al., 2015). Children’s ages at each round ranged from 6 to 18 months (hereafter “1y”, round 1), 4.5 to 5.5 years (“5y”, round 2), 7.5 to 8.5 years (“8y”, round 3) and 11.5 to 12.5 y (“12y”, round 4). Sampling methods have been reported previously (Humphries et al., 2015). Additional study methods are described elsewhere (Barnett, 2012), and are provided at http://www.younglives.org.uk (Young Lives., 2015). From age 1y to age 12y, the YL country cohorts lost between 1.5% and 5.7% of participants to attrition (Ethiopia 114/1999; India 81/2011; Peru 106/2052; Vietnam 36/2000). Household food expenditures were collected starting with round 2; consequently, this study included data from rounds 2, 3 and 4. Children were excluded from each round if they were missing information on anthropometry, rural/urban residence or household food expenditures. Final sample sizes for rounds 2–4, respectively, were: Ethiopia (1744, 1742, 1733); India (1804, 1806, 1801); Peru (1795, 1788, 1775), Vietnam (1788, 1754, 1684).

### 2.2 Study indicators

#### 2.2.1 Household food expenditures

The respondent (usually the mother) was asked to report on consumption of between 21 and 33 food categories in the previous two weeks. For each food group, the respondent estimated (a) total expenditure on all individual foods in that food group consumed in the previous 2 weeks; (b) value of gifts received or food paid in lieu of wages in that food group; and (c) value of own stores used in that food group (whether from the household’s own production, shop or stocks). We aligned food groups across rounds and across countries to generate 18 groups that were included in rounds 2, 3 and 4. We then aggregated food groups in two different ways in preparation for factor analyses. Approach 1: For food groups for which many households reported no expenditures, we aggregated groups into combinations of similar foods to make, for example, a fruit and vegetables group and an animal source foods group (meat, fish, eggs, and dairy). Approach 2: We aggregated food group expenditures to align with the seven food groups in the WHO child dietary diversity measure (World Health Organization Department of Child and Adolescent Health and Development et al., 2010). Based on the YL household census conducted as part of data collection, we generated the number of adult equivalents (AE) in the household (Glewwe and Twum-Baah, 1991) and converted food group expenditures to per AE values. We adjusted food expenditures independently in each country based on country-specific consumer price indices (Young Lives., 2015). These values were adjusted to 2006 local currency and adjusted again to facilitate international comparability using purchasing power parity conversions (Taylor, 2002, Taylor and Sarno, 2004). We summed total food expenditures across food groups to give a total food expenditure per adult equivalent. We winsorized expenditures in each food group to address outliers (Reifman and Keyton, 2010) for each of the four countries, replacing values below the first percentile with the 1st percentile, and replacing values above the 99th percentile with the 99th percentile.

Ethiopia (15.5, 16.2, 17.4) had the lowest total food expenditures per adult equivalent at each time point, and Peru (41.1, 47.3, 55.3) had the highest (Table 1). Ethiopia was the only country with the preponderance of food expenditures in the whole cereals category (Table 1). Peru (10.56–15.48) and Vietnam (8.54–12.0) had similar median levels of expenditures on animal source foods (ASF). In Peru ASF expenditures included dairy, meat and fish and in Vietnam ASF expenditures were predominantly meat and fish. Median egg expenditures were similar in Peru and Vietnam, lower in India, and minimal in Ethiopia (Table 1). The median level of expenditures on ASF in India (3.64–4.23) was about a third of the Peru and Vietnam medians, and the median in Ethiopia (0.59–0.86) was less than a quarter of the Indian expenditures (Table 1). Median fruit and vegetable expenditures were highest in Vietnam (3.35–4.45), followed by Peru (2.39–4.41), India (2.60–3.71) and Ethiopia (0.42–0.56) (Table 1).

**Table 1:** Median household adjusted food group expenditures by round and country (in purchasing power parity adjusted international dollars deflated to 2006).

	5y (n = 1744)	8y (n = 1742)	12y (n = 1733)	
	Median (95% CI)	Median (95% CI)	Median (95% CI)	p value
Ethiopia				
DD^a^: legumes, nuts & seeds	0.94 (0.89, 1.0)	0.88 (0.83, 0.93)	0.98 (0.93, 1.03)	<0.001
DD: starch: pasta/rice/bread	0.00 (0,0)	0 (0,0)	0.81 (0.71, 0.93)	<0.001
DD: starch: whole cereals	7.69 (7.31, 8.00)	7.86 (7.6, 8.1)	7.19 (6.92, 7.39)	<0.001
DD: starch: tubers/roots	0.34 (0.31, 0.37)	0.28 (0.25, 0.30)	0.2 (0.23, 0.29)	<0.001
DD: meat & fish	0.00 (0,0)	0 (0,0)	0.00 (0,0)	<0.001
DD: eggs	0.00 (0,0)	0 (0,0)	0 (0,0)	<0.01
DD: dairy	0.00 (0,0)	0 (0,0)	0.00 (0,0)	<0.01
Total Animal Source Foods (ASF)	0.59 (0.45, 0.74)	0.56 (0.46, 0.66)	0.86 (0.71, 1.07)	<0.001
DD: Fruit & vegetables	0.42 (0.39, 0.45)	0.4 (0.37, 0.42)	0.56 (0.52, 0.59)	<0.001
Salt/spices	0.17 (0.16, 0.18)	0.19 (0.18, 0.20)	0.56 (0.53, 0.60)	<0.001
DD: fats & oils	0.93 (0.88, 0.97)	0.72 (0.69, 0.75)	0.77 (0.74, 0.81)	<0.001
Sugar/honey/sweets	0.46 (0.43, 0.49)	0.44 (0.40, 0.47)	0.47 (0.44, 0.49)	0.80
Prepared foods	0.00 (0,0)	0 (0,0)	0.00 (0,0)	<0.001
Coffee/tea	0.96 (0.92, 1.01)	0.9 (0.87, 0.94)	0.85 (0.82, 0.88)	<0.001
Soft drinks & alcohol	0.00 (0,0)	0 (0,0)	0.00 (0,0)	<0.001
Other foods	0.00 (0,0)	0 (0,0)	0.00 (0,0)	<0.001
Local foods	0.00 (0,0)	0 (0,0)	0.00 (0,0)	0.80
Total household food expenditures	15.52 (15.02, 16.03)	16.16 (15.54, 16.73)	17.42 (16.92, 17.98)	

	5y (n = 1804)	8y (n = 1806)	12y (n = 1801)	
	Median (95% CI)	Median (95% CI)	Median (95% CI)	p value

India				
DD: legumes, nuts & seeds	1.11 (1.08, 1.14)	1.42 (1.37, 1.46)	0.99 (0.96, 1.03)	<0.001
DD: starch: pasta/rice/bread	5.77 (5.62, 5.97)	4.87 (4.65, 5.13)	4.65 (4.42, 4.85)	<0.001
DD: starch: whole cereals	0.00 (0,0)	0.00 (0,0)	0.00 (0,0)	<0.001
DD: starch: tubers/roots	0.28 (0.27, 0.30)	0.33 (0.32, 0.35)	0.30 (0.28, 0.32)	<0.001
DD: meat & fish	1.99 (1.89, 2.12)	2.27 (2.17, 2.40)	2.19 (2.10, 2.30)	<0.001
DD: eggs	0.34 (0.32, 0.36)	0.32 (0.30, 0.34)	0.30 (0.28, 0.32)	0.42
DD: dairy	1.27 (1.16, 1.35)	1.27 (1.20, 1.35)	1.71 (1.63, 1.81)	<0.001
DD: Fruit & vegetables	2.60 (2.50, 2.70)	3.34 (3.21, 3.45)	3.71 (3.59, 3.81)	<0.001
Total Animal Source Foods (ASF)^b^	3.67 (3.50, 3.84)	3.64 (3.49, 3.78)	4.23 (4.07, 4.38)	<0.001
Salt/spices	0.65 (0.63, 0.68)	0.88 (0.84, 0.92)	0.68 (0.66, 0.70)	<0.001
DD: fats & oils	1.68 (1.65, 1.73)	1.57 (1.52, 1.60)	1.62 (1.57, 1.67)	<0.001
Sugar/honey/sweets	1.10 (1.05, 1.14)	1.46 (1.41, 1.52)	0.74 (0.71, 0.79)	<0.001
Prepared foods	0.00 (0,0)	0.30 (0.28, 0.32)	0.00 (0,0)	0.99
Coffee/tea	0.42 (0.41, 0.44)	0.39 (0.38, 0.41)	0.34 (0.33, 0.35)	<0.001
Soft drinks & alcohol	0.00 (0,0)	0.30 (0.19, 0.39)	0.14 (0, 0.38)	0.99
Other foods	0.00 (0,0)	0.00 (0,0)	0.00 (0,0)	0.99
Local foods	0.00 (0,0)	0.00 (0,0)	0.00 (0,0)	0.99
Total household food expenditures	21.34 (20.83, 21.74)	22.24 (21.67, 22.92)	21.02 (20.57, 21.55)	

	5y (n = 1795)	8y (n = 1788)	12y (n = 1775)	
	Median (95% CI)	Median (95% CI)	Median (95% CI)	p value

Peru				
DD: legumes, nuts & seeds	1.19 (1.12, 1.24)	1.38 (1.32, 1.44)	1.51 (1.43, 1.61)	<0.001
DD: starch: pasta/rice/bread	8.82 (8.53, 9.05)	9.15 (8.90, 9.36)	8.94 (8.69, 9.21)	<0.01
DD: starch: whole cereals	1.66 (1.57, 1.82)	2.03 (1.91, 2.15)	2.28 (2.11, 2.43)	<0.001
DD: starch: tubers/roots	2.01 (1.92, 2.08)	2.16 (2.09, 2.28)	2.47 (2.36, 2.58)	<0.001
DD: meat & fish	6.68 (6.34, 7.13)	9.17 (8.57, 9.57)	10.86 (10.54, 11.13)	<0.001
DD: eggs	0.93 (0.88, 0.97)	1.13 (1.08, 1.19)	1.42 (1.37, 1.47)	<0.001
DD: dairy	3.70 (3.48, 3.95)	4.04 (3.81, 4.29)	4.83 (4.62, 5.03)	<0.001
DD: Fruit & vegetables	2.39 (2.29, 2.54)	3.22 (3.10, 3.37)	4.41 (4.18, 4.61)	<0.001
Total Animal Source Foods (ASF)	10.56 (10.08, 11.05)	12.93 (12.51, 13.46)	15.48 (15.01, 16.21)	<0.001
Salt/spices	0.46 (0.44, 0.48)	0.52 (0.49, 0.56)	0.58 (0.55, 0.60)	<0.001
DD: fats & oils	1.24 (1.19, 1.28)	1.47 (1.42, 1.52)	1.35 (1.29, 1.42)	<0.001
Sugar/honey/sweets	1.75 (0.68, 1.83)	1.55 (1.47, 1.62)	1.49 (1.44, 1.56)	<0.001
Prepared foods	0.79 (0.62, 0.96)	1.92 (1.67, 2.21)	4.53 (4.10, 5.11)	<0.001
Coffee/tea	0.41 (0.39, 0.43)	0.40 (0.37, 0.41)	0.46 (0.44, 0.49)	0.02
Soft drinks & alcohol	0.87 (0.82,)0.95	1.05 (0.97, 1.12)	0.91 (0.83, 0.98)	<0.001
Other foods	0.00 (0,0)	0.00 (0,0)	0.00 (0,0)	<0.001
Local foods	0.50 (0.48, 0.53)	0.65 (0.61, 0.68)	0.00 (0,0)	<0.001
Total household food expenditures	41.06 (39.64, 42.28)	47.26 (45.92, 48.58)	55.33 (53.95, 57.17)	

	5y (n = 1788)	8y (n = 1754)	12y (n = 1684)	
	Median (95% CI)	Median (95% CI)	Median (95% CI)	p value

Vietnam				
DD: legumes, nuts & seeds	0.00 (0,0)	0.00 (0,0)	0.00 (0,0)	0.06
DD: starch: pasta/rice/bread	7.94 (7.80, 8.08)	9.12 (8.86, 9.29)	10.02 (9.73, 10.3)	<0.001
DD: starch: whole cereals	0.00 (0,0)	0.00 (0,0)	0.00 (0,0)	<0.001
DD: starch: tubers/roots	0.46 (0.41, 0.49)	0.31 (0.26, 0.35)	0.44 (0.33, 0.51)	<0.001
DD: meat & fish	9.48 (9.12, 9.89)	11.24 (10.79, 11.68)	14.73 (14.23, 15.36)	<0.001
DD: eggs	0.83 (0.79, 0.89)	0.80 (0.75, 0.87)	1.33 (1.29, 1.39)	<0.001
DD: dairy	1.33 (1.10, 1.55)	2.40 (2.22, 2.66)	0.00 (0, 0.79)	<0.001
Total Animal Source Foods (ASF)	8.54 (8.13, 8.96)	10.23 (9.72, 10.68)	12.00 (11.56, 12.58)	<0.001
DD: Fruit & vegetables	3.35 (3.21, 3.47)	3.96 (3.83, 4.12)	4.45 (4.24, 4.67)	<0.001
Salt/spices	0.74 (0.73, 0.76)	1.17 (1.12, 1.22)	1.27 (1.21, 1.31)	<0.001
DD: fats & oils	0.80 (0.78, 0.82)	1.30 (1.24, 1.34)	1.62 (1.57, 1.70)	<0.001
Sugar/honey/sweets	0.95 (0.88, 1.03)	1.10 (1.02, 1.21)	0.68 (0.64, 0.75)	<0.001
Prepared foods	0.00 (0,0)	0.00 (0, 0.85)	0.00 (0,0)	<0.001
Coffee/tea	0.30 (0.26, 0.33)	0.41 (0.35, 0.49)	0.33 (0.22, 0.46)	<0.01
Soft drinks & alcohol	0.00 (0,0)	0.54 (0.45, 0.62)	0.57 (0.42, 0.67)	<0.001
Other foods	0.00 (0,0)	0.00 (0,0)	0.00 (0,0)	<0.001
Local foods	0.00 (0,0)	0.00 (0,0)	0.00 (0,0)	1.0
Total household food expenditures	32.72 (31.58, 33.76)	40.79 (39.43, 42.05)	44.72 (43.00, 45.82)	

All expenditure figures are per adult equivalent in the household.

a DD indicates that a food group is one of the seven dietary diversity food groups used in this analysis.

b Total ASF is the sum of meat & fish, eggs and dairy.

#### 2.2.2 Child anthropometry

Field workers measured height using locally-made stadiometers with standing plates and moveable head boards accurate to 1 mm. We calculated HAZ using WHO 2006 standards for children 0–59 months (de Onis et al., 2006) and WHO 2007 standards for older children (de Onis et al., 2007). Field workers measured weight using calibrated digital balances (Soehnle) with 100 g precision. We calculated body-mass-indices for age (BMI-Z) using WHO growth curves. All measurements were taken according to WHO guidelines (World Health Organization Department of Nutrition for Health and Development and Development, 2008, United Nations Department of Technical Co-operation for Development and Statistical Office, 1986). Birth dates were drawn from children’s health cards when available, and mothers’ reports otherwise. Our analyses focused on HAZ and BMI-Z as the standard indicators of malnutrition in children (Black et al., 2013b).

#### 2.2.3 Dietary diversity

We assessed individual dietary diversity by summing the number of standard dietary diversity food groups that the child was reported to have eaten the previous day (World Health Organization Department of Child and Adolescent Health and Development et al., 2010). We combined food groups into the seven recommended categories at age 5 y, including (1) starches (cereals, roots and tubers), (2) meat (meat, fish), (3) eggs, (4) legumes and nuts, (5) dairy, (6) fruit and vegetables, and (7) fats and oils. At ages 8 y and 12 y, vitamin A rich fruits and vegetables were recorded as separate categories as in the adult version of dietary diversity.

#### 2.2.4 Control variables

Other measures included round of data collection, sex of child, and rural/urban residence.

### 2.3 Statistical methods

We used Stata (version 14.0, 2013. Stata Corp) for all analyses. Results were considered statistically significant for p values < 0.05. We present associations between household food expenditures, dietary diversity and child growth (HAZ, BMI-Z) using (a) total household food expenditures and (b) factor analysis extracted from household expenditures on the dietary diversity food groups, controlling for total food expenditures, round of data collection, rural/urban residence and sex of child.

Factor analysis: we pooled food expenditures at ages 5y, 8y and 12y in each country then analyzed food expenditures at the country level. We ran both factor analysis and PCA with one, two and three factors for each country, with the upper limit of three factors based on scree plots and eigen values >1.0. We assessed each PCA and factor solution for loading >0.40 for at least three food groups on each component or factor. None of the PCA solutions in any of the four countries met those criteria. The one-factor solution in all four countries met these requirements, so we utilized the one-factor solution for this analysis. The one-factor solution represents the latent driver of households’ allocations of their food finances across food groups, and we refer to the latent variable as the ‘household food group expenditure index’ (HFGEI), which represents food choices within the constraints of household preferences, household resources, and local food pricing and availability.

We used multivariable ordinary least squares regressions for HAZ and BMI-Z to examine associations between food expenditures and anthropometry.

## 3 Results

Mean HAZ increased from ages 5y to 12y in India (−1.65 to −1.45), Peru (−1.53 to −0.97) and Vietnam (−1.35 to −1.06), although it remained constant for Ethiopia (−1.48 to −1.47) (Table 2). BMI-Z decreased from ages 5y to 12y in Ethiopia (−0.62 to −1.82), India (−1.16 to −1.35) and Vietnam (−0.31 to −0.65). Mean HAZ was negative in all countries across all rounds, and mean BMI-Z was negative across all rounds in all countries except Peru, where mean BMI-Z ranged from 0.52 to 0.67 (Table 2). The percentages of individuals who lived in rural areas remained relatively constant in Ethiopia (59–60%), India (72–74%) and Vietnam (80–81%), although Peru experienced a decrease from 45% to 27% because of migration to cities (Table 2). In all countries there was an increase in mean dietary diversity from age 5y to age 8y, and a decrease from age 8y to age 12y (Table 2). Peru had the highest dietary diversity at each round, and Ethiopia had the lowest.

**Table 2:** Child and household characteristics by round and country.

	5y (n = 1744)	8y (n = 1742)	12y (n = 1733)
	Mean or% (95% CI)	Mean or% (95% CI)	Mean or% (95% CI)
Ethiopia			
Anthropometry			
HAZ	−1.48	−1.22	−1.47
BMI-Z	−0.62	−1.28	−1.82

Background characteristics			
Female (%)	46.7	46.7	46.7
Rural residence (%)	60.0	60.1	59.3
Dietary diversity	3.65	3.89	3.43

	5y (n = 1804)	8y (n = 1806)	12y (n = 1801)

India			
Anthropometry			
HAZ	−1.65	−1.46	−1.45
BMI-Z	−1.16	−1.39	−1.35

Background characteristics			
Female (%)	46.7	46.6	46.5
Rural residence (%)	74.1	73.3	72.1
Dietary diversity	4.33	4.49	3.84

	5y (n = 1795)	8y (n = 1788)	12y (n = 1775)

Peru			
Anthropometry			
HAZ	−1.53	−1.15	−0.97
BMI-Z	0.67	0.52	0.54

Background characteristics			
Female (%)	49.9	50.1	49.8
Rural residence (%)	45.1	23.2	26.7
Dietary diversity	5.25	6.23	5.86

	5y (n = 1788)	8y (n = 1754)	12y (n = 1684)

Vietnam			
Anthropometry			
HAZ	−1.35	−1.11	−1.06
BMI-Z	−0.31	−0.70	−0.65

Background characteristics			
Female (%)	48.7	48.7	48.6
Rural residence (%)	80.3	80.8	81.3
Dietary diversity	4.94	5.29	4.42 (4.35, 4.48)

**Fig. 2:**
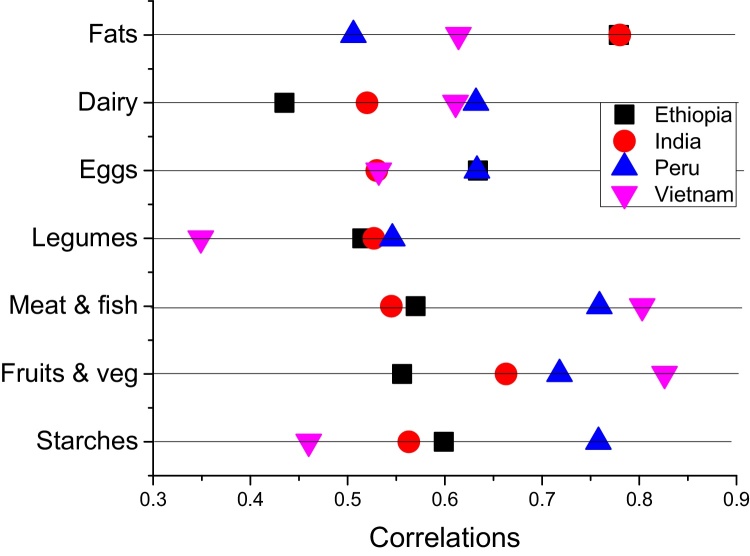
Correlations between Household Food Group Expenditure Index and Child Dietary Diversity Food Groups.

Five food groups loaded on the first factor, HFGEI, in all of the countries (starches, fruit and vegetables, meat and fish, eggs, and fats), contributing significantly to the common variance across all of the food groups (Table 3). However, we found that relative loadings for each of the food groups were quite different across countries. Correlations of HFGEI with food group expenditures also varied across the four countries (Fig. 2). For example, the correlation between meat and fish expenditures and HFGEI were lowest in India (0.54), slightly higher in Ethiopia (0.57), and highest in Peru (0.76) and Vietnam (0.80).

**Table 3:** Dietary diversity food group loadings on HFGEI, by country.

	Ethiopia		India		Peru		Vietnam	
	HFGEI	Uniqueness	HFGEI	Uniqueness	HFGEI	Uniqueness	HFGEI	Uniqueness
Starches	0.497	0.753	0.466	0.783	0.668	0.554	0.397	0.842
Fruits and vegetables	0.461	0.788	0.549	0.698	0.633	0.6	0.714	0.491
Meat and fish	0.472	0.777	0.451	0.796	0.669	0.553	0.694	0.519
Legumes	0.427	0.818	0.436	0.81	0.481	0.769	0.302	0.909
Eggs	0.525	0.725	0.439	0.807	0.558	0.689	0.459	0.789
Dairy	0.36	0.87	0.431	0.814	0.557	0.699	0.528	0.721
Fats	0.645	0.583	0.646	0.583	0.446	0.801	0.531	0.718
Eigenvalue	1.686		1.708		2.345		2.01	
Proportion	1.193		1.256		1.123		1.191	

Households in the lowest quartile of HFGEI in Ethiopia spent on average 57% of their food expenditures on starches (Table 4). Adjusted expenditure values allow cross-country comparisons. Based on mean percentages of total food expenditures on specific food groups, we present a picture of how households allocate their food budget. Across all four countries, mean percentages of food expenditures on meat and fish were lowest for households in the lowest quartile of HFGEI, and highest in the highest quartile of HFGEI (Table 4). We observed the same pattern for fruit and vegetable expenditures for Peru and Vietnam, although in Ethiopia and India there were minimal changes in percentage expenditures on fruit and vegetables across the four quartiles of HFGEI (Table 4).

**Table 4:** a) Percent food group expenditures on dietary diversity food groups by HFGEI quartiles, by country. b) Median food group expenditures (in purchasing power parity adjusted international dollars per adult equivalent) on dietary diversity food groups by HFGEI quartiles, by country.

	Quartile 1	Quartile 2	Quartile 3	Quartile 4	p value*
a)					
Ethiopia					
Starches	57.0	60.0	57.0	48.1	<0.001
Fruit and vegetables	2.6	3.0	2.8	3.2	<0.001
Meat	0	0	0	5.5	<0.001
Legumes, nuts, and seeds	4.0	6.0	6.0	5.6	<0.001
Eggs	0	0	0	1.4	<0.001
Dairy	0	0	0	3.0	<0.001
Oil and oil seed	3.9	4.8	5.0	6.1	<0.001

India					
Starches	25.9	28.5	28.2	26.4	<0.001
Fruit and vegetables	15.3	15.0	15.0	15.1	<0.001
Meat	8.8	10.0	10.8	11.3	<0.001
Legumes, nuts, and seeds	5.8	5.6	5.4	5.0	<0.001
Eggs	0	1.5	1.5	1.6	<0.001
Dairy	4.6	7.5	7.5	8.5	<0.001
Oil and oil seed	7.4	7.6	7.6	7.7	<0.001

Peru					
Starches	35.4	31.9	29.1	26.5	<0.001
Fruit and vegetables	5.6	6.6	7.1	8.4	<0.001
Meat	14.0	18.2	20.5	21.4	<0.001
Legumes, nuts, and seeds	2.6	2.9	2.8	2.9	<0.001
Eggs	2.1	2.4	2.5	2.6	<0.001
Dairy	6.4	8.4	9.8	9.9	<0.001
Oil and oil seed	3.4	3.1	2.8	2.5	<0.001

Vietnam					
Starches	37.0	27.8	22.9	17.5	<0.001
Fruit and vegetables	8.6	9.6	10.4	11.9	<0.001
Meat	26.3	29.4	29.9	29.7	<0.001
Legumes, nuts, and seeds	0	0	0	0	<0.001
Eggs	1.2	2.4	2.6	2.6	<0.001
Dairy	0	2.8	5.7	5.7	<0.001
Oil and oil seed	3.2	3.0	2.9	2.9	<0.001

^b)^					
Ethiopia					
Starches	4.89	8.21	10.82	14.90	<0.001
Fruit and vegetables	0.22	0.35	0.56	0.99	<0.001
Meat	0	0	0	1.77	<0.001
Legumes, nuts, and seeds	0.33	0.8	1.19	1.77	<0.001
Eggs	0	0	0	0.47	<0.001
Dairy	0	0	0	0.96	<0.001
Oil and oil seed	0.36	0.69	1.08	1.93	<0.001

India					
Starches	3.25	5.29	6.62	8.88	<0.001
Fruit and vegetables	1.95	2.77	3.51	3.51	<0.001
Meat	1.15	1.87	2.52	2.52	<0.001
Legumes, nuts, and seeds	0.74	1.04	1.28	1.28	<0.001
Eggs	0	0.28	0.37	0.37	<0.001
Dairy	0.62	1.17	1.74	1.74	<0.001
Oil and oil seed	0.95	1.46	1.82	1.82	<0.001

Peru					
Starches	8.94	12.69	15.52	20.96	<0.001
Fruit and vegetables	1.46	2.70	3.90	6.82	<0.001
Meat	3.67	7.40	10.98	16.99	<0.001
Legumes, nuts, and seeds	0.69	1.19	1.54	2.39	<0.001
Eggs	0.56	0.97	1.38	2.06	<0.001
Dairy	1.72	3.43	5.27	7.99	<0.001
Oil and oil seed	0.87	1.25	1.50	2.03	<0.001

Vietnam					
Starches	7.71	9.32	10.71	12.57	<0.001
Fruit and vegetables	1.86	3.26	4.98	9.13	<0.001
Meat	5.55	9.86	14.34	22.43	<0.001
Legumes, nuts, and seeds	0	0	0	0	<0.001
Eggs	0.30	0.82	1.28	1.89	<0.001
Dairy	0	0.99	2.79	6.72	<0.001
Oil and oil seed	0.69	1.01	1.44	2.07	<0.001

* p values indicate the probability that observed differences in median expenditures across HFGEI quartiles in each country are due to chance.

Our findings suggest that total food expenditures were positively associated with HAZ (p < 0.05) and BMI-Z (p < 0.05) in all four countries after adjusting for study round, rural residence, and whether the child was female (Table 5). With adjustments for dietary diversity and HFGEI, total food expenditures were no longer a significant predictor of HAZ in Ethiopia and India. HFGEI was associated with significant increases in child HAZ in Ethiopia, India, and Vietnam with adjustments for data collection round, total food expenditures, dietary diversity, rural residence and whether the child was female (Table 5).

**Table 5:** Child anthropometry (HAZ & BMI-Z) in relation to Household Food Expenditures, by Country.

	HAZ				BMI-Z			
	Coefficient	p value	Coefficient	p value	Coefficient	p value	Coefficient	p value
Ethiopia								
Model R^2^	0.0624		0.0656		0.203		0.207	
Age 8y	0.254	<0.001	0.256	<0.001	−0.66	<0.001	−0.652	<0.001
Age 12y	−0.009	0.80	−0.003	0.94	−1.2	<0.01	−1.19	<0.001
Total food expenditures^a^	0.228	<0.001	0.093	0.15	0.102	<0.001	−0.113	0.07
HFGEI^a^			0.067	0.03			0.108	<0.001
Dietary diversity^a^			0.037	0.05			0.033	0.08
Rural	−0.393	<0.001	−0.363	<0.001	−0.224	<0.001	−0.187	<0.001
Female			0.073	0.01			−0.073	<0.01

India								
Model R^2^	0.066		0.076		0.032		0.037	
Age 8y	0.184	<0.001	0.174	<0.001	−0.235	<0.001	−0.23	<0.001
Age 12y	0.198	<0.001	0.207	<0.001	−0.185	<0.001	−0.204	<0.001
Total food expenditure	0.232	<0.001	−0.067	0.27	0.183	<0.001	0.15	0.04
HFGEI			0.141	<0.001			0.023	0.46
Dietary diversity			0.071	<0.001			−0.048	0.04
Rural	−0.509	<0.001	−0.487	<0.001	−0.394	<0.001	−0.399	<0.001
Female			0.094	0.001			0.155	<0.001

Peru								
Model R^2^	0.093		0.095		0.034		0.053	
Age 8y	0.212	<0.001	0.165	0.003	−0.223	<0.001	−0.187	<0.001
Age 12y	0.337	<0.001	0.325	<0.001	−0.23	<0.001	−0.208	<0.001
Total food expenditure	0.117	<0.001	0.094	0.01	0.061	<0.001	0.108	<0.001
HFGEI			0.016	0.68			−0.047	0.06
Dietary diversity			0.075	0.005			−0.047	<0.01
Rural	−0.903	<0.001	−0.883	<0.001	−0.363	<0.001	−0.361	<0.001
Female			0.025	0.56			−0.272	<0.001

Vietnam								
Model R^2^	0.1437		0.159		0.109		0.117	
Age 8y	0.165	<0.001	0.141	<0.001	−0.439	<0.001	−0.44	<0.001
Age 12y	0.161	<0.001	0.228	<0.001	−0.434	<0.001	−0.461	<0.001
Total food expenditure	0.234	<0.001	0.147	<0.001	0.169	<0.001	0.064	0.05
HFGEI			0.065	0.033			0.133	<0.001
Dietary diversity			0.149	<0.001			0	0.99
Rural	0.514	<0.001	−0.479	<0.001	−0.628	<0.001	−0.631	<0.001
Female			0.034	0.21			−0.188	<0.001

a Total Food Expenditures, Household Food Group Expenditure Index and dietary diversity are standardized in units of standard deviation.

In the fully-adjusted models that included study round, HFGEI, dietary diversity, rural residence, and whether the child was female, total food expenditures remained significantly associated with BMI-Z in India, Peru and Vietnam. Dietary diversity was inversely associated with BMI-Z in India and Peru; namely a higher diversity score was associated with lower BMI-Z. Coefficients for both countries were essentially the same (−0.048 for India and −0.047 for Peru) as were p values (0.04 and <0.01). It is important to note that mean BMI-Z in India was negative at all three ages, and mean BMI-Z in Peru was positive. Total food expenditures were not significantly associated with BMI-Z in Ethiopia. In Peru, the only country with a positive mean BMI-Z, there was a significant positive association between total food expenditures and BMI-Z.

## 4 Discussion

We examined data from Young Lives, a cohort of children from Ethiopia, India, Peru and Vietnam, spanning three ages (5, 8 and 12 yrs), to assess whether total food expenditures (dietary quantity), dietary diversity (dietary quality), and household food group expenditure patterns were associated with measures of undernutrition. We found that total food expenditures were positively associated with HAZ (p < 0.05) and BMI-Z (p < 0.05) in all four countries with adjustments for study round, rural residence, and whether the child was female. Similar to other studies (Campbell et al., 2010, Sari et al., 2010, Torlesse et al., 2003, Mauludyani et al., 2014, Rosinger et al., 2013), we found associations between specific food expenditure patterns and nutritional status. For example, Rosinger and colleagues (Rosinger et al., 2013) note that in a nomadic Amazonian population, men (but not women) living in households with high monetary expenditures on market foods (top third) had significantly higher BMI, weight, percentage body fat, and probability of being overweight or obese. However, their study focused on adults, not children. A recent analysis of household food expenditures and inflation in Mozambique during 2008/2009 (Arndt et al., 2016) found higher rates of acute child malnutrition (lower weight-for-age z scores, WAZ), in periods with higher quarterly rates of inflation.

When we included dietary diversity and the HFGEI in multivariate HAZ models, total food expenditures were not significant predictors of HAZ in Ethiopia and India. According to these results, how households allocate expenditures across food groups, *may* be a more important driver of child linear growth than total food expenditures, at least for Ethiopia and India. In multivariate BMI-Z models that included study round, HFGEI, dietary diversity, rural residence, and whether the child was female, total food expenditures remained significantly positively associated with BMI-Z in India, Peru and Vietnam.

We found that dietary diversity was inversely associated with BMI-Z in India and Peru. In Peru, where mean BMI-Z is positive, the inverse relationship suggests that higher dietary diversity is associated with a lower risk of higher BMI-Z, and potential overweight. In India, where mean BMI-Z is negative, the inverse association between dietary diversity and BMI-Z is more challenging to interpret. Previous analysis by this team found that dietary diversity was not a significant mediator between food security and child anthropometry in India (Humphries et al., 2015), although another recent study concluded that poor dietary diversity is an important predictor of chronic undernutrition in India (Corsi et al., 2016). The HFGEI was associated with significant increases in child HAZ in Ethiopia, India, and Vietnam with adjustments for data collection round, total food expenditures, dietary diversity, rural residence and whether the child was female. All countries showed an increase in mean dietary diversity from age 5y to age 8y, which is likely an artifact of the addition of one additional food group (vitamin A rich fruits and vegetables) at age 8y, and a decrease from age 8y to age 12y with the same number of food groups in those two rounds.

Five food groups loaded on HFGEI in all countries (starches, fruit and vegetables, meat and fish, eggs, and fats), contributing significantly to the common variance across all of the food groups, though relative loadings for each of the food groups were quite different. While a number of researchers (Becquey et al., 2010, Bedard et al., 2015, Devlin et al., 2012) have used factor analysis, PCA, and other approaches to identify groups of *foods* based on dietary intake data, use of household food *expenditure* data is less common (Campbell et al., 2010, Sari et al., 2010, Torlesse et al., 2003). In their examination of the relationship between food expenditures and nutritional status, Rosinger and colleagues identified five categories of market foods in a nomadic Amazonian population. These foods are known to reflect the nutrition transition and included dairy, oils, market meats, refined carbohydrates, and sweets. However, Rosinger et al. (Rosinger et al., 2013) did not conduct factor analysis but rather summed total expenditures for each food group. In contrast, Fan and colleagues (Fan et al., 2007) found eight clusters of food expenditures ranging from “balanced” meals eaten largely at home to “fast food” and “full service” meals consumed outside the household.

## 5 Limitations

Our study had several limitations. The intra-household allocation of food is unspecified, so we do not know the relationship between household food expenditures and food consumption of individual children. YL obtained information on child dietary diversity by asking the mother or caregiver at ages 5y and 8y, and by asking the child directly at 12y. In order to fully interpret the relationship between maternal vs. self-reported dietary diversity for children we need to better understand patterns of dietary diversity as children age. This study focuses primarily on current food consumption as a predictor of child growth, although extensive literature (Dangour et al., 2013), including several recent studies, have noted the importance of non-food influences on child height (Griffen, 2016, Krishna et al., 2016, Puentes et al., 2016, Dearden, et al. 2017).

As noted previously, the challenges associated with using dietary intake data are numerous and include the importance of research staff with dietary data expertise, substantial interview time, the difficulty of capturing such information, and complex analytic methods. The conversion of food expenditure data into estimates of nutrient intake requires details about food items purchased, local and seasonal costs and regularly updated country-specific tables of food composition. In addition, assumptions regarding intra-household food distribution are required. Use of food expenditure data as an indicator of latent household food group expenditure patterns is simpler, though still requiring assumptions about intra-household distribution, and our study indicates that this measure provides insight into dietary patterns that are associated with child growth. Importantly, food choice patterns may reflect a modifiable component of household behavior, which could be addressed through behavior-change or market price interventions.

## 6 Conclusions

In this study, we 1) provide a new way to consider food preferences, relative prices and availabilities by observing household food group expenditure patterns that are relevant for nutritional status, 2) contribute to the literature on how household dietary quantity and quality predict undernutrition, and 3) demonstrate the utility of food group expenditure data as an indicator of potential nutrition risk.

We used factor analysis to identify an underlying pattern of consumption across food groups that is related to child growth. Factor analysis and other data reduction techniques may add important information over and above what is provided by total food expenditures alone (as reflected in their significance in our estimates). This is useful because although disaggregated expenditure data may be an important proxy of the types of foods households consume, in the aggregate, their complexity may pose a challenge for policy makers, program planners, and managers seeking to understand associations between food expenditures and children’s nutritional status.

This is particularly important because household expenditure surveys are routinely conducted in many low- and middle-income countries. These surveys provide an important but often neglected source of information about how much money households allocate to food, which foods they prioritize, and whether such prioritization provides a diversity of healthy foods. Thus, household expenditure surveys may also shed light on children’s growth.

More studies, especially those that are longitudinal in nature, are needed to validate our findings and further explore changes in dietary diversity as children age. We intend to continue examining Young Lives data to explore potential longitudinal effects, sibling effects, and the relationship of HFGEI to other household characteristics such as parental schooling attainment.

In summary, our study has shown the potential for using disaggregated household food expenditure data to explore patterns of household food consumption as predictors of child growth. We report significant differences in these patterns across four diverse low- and middle-income countries.

## Conflict of interest

Mary E. Penny has received research funding from the food industry for studies unrelated to this research. The other authors declare no conflict of interest.

## Funding

This study is based on research funded by the Bill & Melinda Gates Foundation (Global Health Grant OPP1032713), Eunice Shriver Kennedy National Institute of Child Health and Development (Grant R01 HD070993) and Grand Challenges Canada (Grant 0072-03). The study uses data from Young Lives, a 15-year survey investigating the changing nature of childhood poverty in Ethiopia, India (Andhra Pradesh and Telangana), Peru and Vietnam (www.younglives.org.uk). Young Lives is core-funded by the UK Department for International Development (DFID) and was co-funded from 2010 to 2014 by the Netherlands Ministry of Foreign Affairs. The authors are responsible for all the findings and conclusions: they do not necessarily reflect positions or policies of the Bill & Melinda Gates Foundation, the Eunice Shriver Kennedy National Institute of Child Health and Development, Grand Challenges Canada, Young Lives, DFID or other funders.

## Ethical review

The University of Oxford Ethics Committee and the Peruvian Instituto de Investigación Nutricional IRB approved YL study protocols. Approval for these analyses was obtained from the University of Pennsylvania. Written parental consent was obtained at the beginning of the study and confirmed verbally at each round. Assent was obtained from children.
