# Supplementary material for: Household food group expenditure patterns are associated with child anthropometry at ages 5, 8 and 12 years in Ethiopia, India, Peru and Vietnam
Source: Econ Hum Biol. 2017 Aug;26:30–41. doi: 10.1016/j.ehb.2017.02.001 (PMC5555831; doi:10.1016/j.ehb.2017.02.001)
Supplement: Supplementary file 1 [file mmc1.docx]

**Appendix: Ethical review**

The University of Oxford Ethics Committee and the Peruvian Instituto de Investigación Nutricional IRB approved YL study protocols. Approval for these analyses was obtained from the University of Pennsylvania. Written parental consent was obtained at the beginning of the study and confirmed verbally at each round. Assent was obtained from children.
